# Widely targeted metabolomic analysis reveals that volatile metabolites in cigar tobacco leaves dynamically change during fermentation

**DOI:** 10.1016/j.bbrep.2023.101532

**Published:** 2023-08-19

**Authors:** Jiaohong Fan, Guanghui Kong, Heng Yao, Yuping Wu, Gaokun Zhao, Fuling Li, Guanghai Zhang

**Affiliations:** a College of Tobacco, Yunnan Agriculture University, Kunming, Yunnan, 650201, China; b Yunnan Academy of Tobacco Agricultural Sciences, Kunming, Yunnan, 650021, China

**Keywords:** Cigar tobacco leaves, Fermentation, Volatile metabolites, Metabolic pathway, Flavor

## Abstract

Changes in volatile metabolites during cigar tobacco leaves fermentation as well as the metabolic pathways of metabolites with significant differences were investigated to determine the influence of cigar tobacco leaves fermentation on its flavor. The volatile substances in cigar tobacco leaves at different stages were detected by headspace-solid phase microextraction gas chromatography-mass spectrometry (HS-SPME-GC-MS), and the main differences in volatile substances in cigar tobacco leaves at different fermentation stages of Yunxue1 in Yuxi production area were analyzed by principal component analysis (PCA) and orthogonal partial least squares discriminant analysis (OPLS-DA). The results show that in the process of cigar tobacco leaves fermentation (YXF0, YXF1, YXF2, YXF3, YXF4, YXF5), a total of 613 volatile metabolites were detected, and a significant difference was found in 263 kinds of metabolites. Among them, the main upregulated differential metabolites were 1,3,6,10-Cyclotetradecatetraene, 3,7,11-trimethyl-14-(1-methylethyl)-, [*S*-(E,Z,E,E)]-, Benzoic acid, Benzaldehyde, etc. While the main downregulated differential metabolites included beta.-Myrcene, *trans*-Farnesol, etc. The metabolites with significant differences are mainly concentrated in the biosynthesis of monoterpenes, diterpenes, sesquiterpenes and triterpenes, the degradation metabolism of amino acids, such as valine, leucine and isoleucine, and the biosynthesis of phenylpropyl. There were 8 different metabolites in 5 groups, including 4- (1-methylethyl) -1-cyclohexene-1-formaldehyde、2, 4-dihydroxyacetophenone、2-methylbutyl 3-methylbutyrate and methylpyrazine, all of which showed upregulation trend during fermentation. In the fermentation process, volatile metabolites participate in various synthesis and degradation pathways. The biosynthesis pathway of terpenes and amino acid synthesis and degradation pathway are connected to produce various terpenes, aldehydes and other substances, such as 1,3,6,10-Cyclotetradecatetraene, 3,7,11-trimethyl-14-(1-methylethyl)-, [*S*-(E,Z,E,E)]-、benzaldehyde and 4-hydroxybenzaldehyde, which are conducive to the overall flavor and quality of cigar tobacco leaves.

## Highlights

• The terpenoids and esters are the main differential metabolites in fermentation.
• The main upregulated differential metabolites were benzaldehyde, palmitoleic acid, 4-hydroxybenzaldehyde.
• The main downregulated differential metabolites included beta.-Myrcene, α-pinene and β-pinene.
• The typical formation of cigar flavor is determined by degradation metabolism of amino acids and biosynthesis of terpenoids.

## 1 Introduction

A cigar generally contains fresh tobacco leaves that have underwent a series of treatments, such as drying, fermentation, and aging; in addition, other artificial cigarette rolls are a significant tobacco product and are composed of wrapper, binder and filler, of which the innermost is the filler, rolled in the outside of the filler is the binder, wrapped in the outermost is the wrapper [1]. Compared to ordinary cigars, artificial cigars cause less harm to the human body; in addition, these cigars exhibit a sufficient aroma, good aroma temperament and sweet taste [2]. Foreign cigar production has a long history, planting methods and scale are relatively stable, and there are stable cigar raw materials source and guarantee [3]. Research shows that [4], the domestic handmade cigar market sales from 5.48 million in 2019 rapidly broke through to 20 million in 2021, and the development of domestic cigars has reached a new historical starting point. In recent years, cigar consumption has increased rapidly in developed areas of our country and shows good prospects for market development [5]. In cigar tobacco leaves production, the quality of tobacco raw materials is affected by variety [6,7], cultivation, drying [8,9], fermentation and other factors. Among these factors, fermentation is the main link affecting the improvement in cigar tobacco leaves quality. The appearance quality, physical characteristics, chemical composition and sensory quality of tobacco leaves can be effectively improved by proper fermentation technology. Fermentation has a great impact on the homogenization production and quality improvement of cigar tobacco leaves, which can reduce some quality defects of the original tobacco, increase the potency of tobacco aroma, and significantly enhance the smoking quality [10]. Fermentation is a processing method used to improve the availability and sensory quality of tobacco leaves. It is divided into two categories, natural fermentation and artificial fermentation.

The chemical composition of tobacco is complex and diverse, and more than 3000 kinds have been identified, including carbohydrates, nitrogen compounds, polyphenols, pigments, organic acids and ketones. The chemical composition of tobacco is the fundamental factor that determines the quality of tobacco [11]. Fermentation can change the chemical composition of tobacco and is an important means to improve the quality of tobacco processing. Unfermented tobacco is heavily impure, is an irritant, causes uncoordinated aroma and rough smoke defects and cannot be directly used for the production of cigarettes and other tobacco products [12]. Different fermentation methods exert different effects on tobacco quality improvement. Appropriate fermentation processes can greatly improve tobacco quality and improve the economic benefits of tobacco production [13]. The organic acids in tobacco are mainly dibasic acid and ternary acid, and the content of monic acid is very small. The two and ternary acids are stearic acid, palmitic acid, malic acid and linoleic acid [14].

The aroma components of cigar tobacco leaf mainly originate from the tobacco leaf and the fermentation process. The analysis and evaluation of the aroma components of cigar tobacco leaves can directly reflect the composition of raw materials of cigar tobacco leaves and the characteristics of fermentation technology and can provide an important basis for the production and quality control of cigar tobacco leaves [15,16]. There are many kinds of aromatic substances in tobacco leaves, and the content of most of them is very low. The aroma substances in tobacco leaves include aroma substances and aroma precursor substances [17]. These aroma precursors are transformed and degraded into aroma substances that can directly affect the flavor of flue-cured tobacco during the growth, maturation, modulation and aging of tobacco leaves [18]. Carotenoids can be degraded into important aroma compounds, such as ketones [19]. Tobacco phenolic substances, including phenols and their derivatives, play an important role in tobacco growth and modulation and are important indicators used to evaluate the quality of tobacco leaves [20]. According to the aromatic functional groups, they can be divided into ketones, aldehydes, acids, esters, alcohols and alkanes. Esters are important aroma precursors in tobacco. Siberanoids are important terpenoids in tobacco. The oxidative fracture of the carbon chain of siberanoid compounds directly produces aldehydes and ketones, such as solanone, geranylacetone and solanedione [21].

At present, research on cigar mainly focuses on tobacco leaves preparation [22,23] and fermentation methods [24,25], but there are few studies on the change in volatile substances in cigar tobacco leaves fermentation, the enrichment pathway of metabolites with significant differences and the influence of volatile metabolites on cigar flavor during fermentation. Therefore, by studying the changes in volatile metabolites in the fermentation process of cigar tobacco leaves and the main enrichment pathways of metabolites with significant differences, this paper revealed the internal mechanism of fermentation on cigar flavor. Thus, the research provides a scientific basis for optimizing high-quality cigar tobacco leaves fermentation technology in the future and lays a solid foundation to produce of high-quality cigar tobacco leaves raw materials.

## 2 Materials and methods

### 2.1 Plant materials and experimental design

The cigar tobacco grown in Yuxi (YX) was selected and sorted after drying, and the fermentation test was carried out according to the agricultural fermentation technology of cigar tobacco. The specific technical requirements of agricultural fermentation are as follows: the tobacco leaves sorted according to type and position after being prepared are bundled and humidified to increase the moisture content of tobacco leaves. After the tobacco leaves are placed until the moisture meets the fermentation requirements, the ratio of length, width and height is about 2:1:1. When the fermentation temperature reaches the set temperature, the stack is turned over four times, and when the stack temperature and tobacco leaf moisture content meet the requirements, the stack is unpacked. Samples were taken from the upper, middle and lower layers of the tobacco stack before fermentation, each stacking and the end of fermentation and unstacking. The tobacco leaves samples obtained at each period were labeled as YXF0, YXF1, YXF2, YXF3, YXF4 and YXF5, and there were three replicates in each group.

### 2.2 Sample preparation and extraction

Materials were harvested, weighted, immediately frozen in liquid nitrogen, and stored at -80 °C until needed. Samples were ground to a powder in liquid nitrogen.

500 mg (1 mL) of the powder was transferred immediately to a 20 mL head-space vial (Agilent, Palo Alto, CA, USA), containing NaCl saturated solution, to inhibit any enzyme reaction. The vials were sealed using crimp-top caps with TFE-silicone headspace septa (Agilent). At the time of SPME analysis, each vial was placed in 60 °C for 5 min, then a 120 μm DVB/CWR/PDMS fibre (Agilent) was exposed to the headspace of the sample for 15 min at 60 °C.2.3 GC-MS conditions for metabolomics analysis.

### 2.3 GC-MS conditions for metabolomics analysis

After sampling, desorption of the VOCs from the fibre coating was carried out in the injection port of the GC apparatus (Model 8890; Agilent) at 250 °C for 5 min in the splitless mode. The identification and quantification of VOCs was carried out using an Agilent Model 8890 GC and a 7000D mass spectrometer (Agilent), equipped with a 30 m × 0.25 mm × 0.25 μm DB-5MS (5% phenyl-polymethylsiloxane) capillary column. Helium was used as the carrier gas at a linear velocity of 1.2 mL/min. The injector temperature was kept at 250 °C and the detector at 280 °C. The oven temperature was programmed from 40 °C (3.5 min), increasing at 10 °C/min to 100 °C, at 7 °C/min to 180 °C, at 25 °C/min to 280 °C, hold for 5 min. Mass spectra was recorded in electron impact (EI) ionisation mode at 70 eV. The quadrupole mass detector, ion source and transfer line temperatures were set, respectively, at 150, 230 and 280 °C. The MS was selected ion monitoring (SIM) mode was used for the identification and quantification of analytes.

### 2.4 Data analysis of metabolites in cigar tobacco leaves

Unsupervised PCA (principal component analysis) was performed by statistics function prcomp within R (www.r-project.org). The data was unit variance scaled before unsupervised PCA. The HCA (hierarchical cluster analysis) results of samples and metabolites were presented as heatmaps with dendrograms, while pearson correlation coefficients (PCC) between samples were calculated by the cor function in R and presented as only heatmaps. Both HCA and PCC were carried out by R package ComplexHeatmap. For HCA, normalized signal intensities of metabolites (unit variance scaling) are visualized as a color spectrum.

### 2.5 Differential metabolites selected in cigar tobacco leaves

For two-group analysis, differential metabolites were determined by variable importance in projection (VIP, VIP ≥1) and absolute Log_2_FC (|Log_2_FC| ≥ 1.0). VIP values were extracted from OPLS-DA result, which also contain score plots and permutation plots, was generated using R package MetaboAnalystR. The data was log transform (log2) and mean centering before OPLS-DA. In order to avoid overfitting, a permutation test (200 permutations) was performed.

### 2.6 KEGG annotation and enrichment analysis of differential metabolites

Identified metabolites were annotated using KEGG Compound database (http://www.kegg.jp/kegg/compound/), annotated metabolites were then mapped to KEGG Pathway database (http://www.kegg.jp/kegg/pathway.html). Pathways with significantly regulated metabolites mapped to were then fed into MSEA (metabolite sets enrichment analysis), their significance was determined by hypergeometric test's p-values.

## 3 Results and analysis

### 3.1 Metabolite composition and principal component analysis of cigar tobacco leaves

**Fig. 1:**
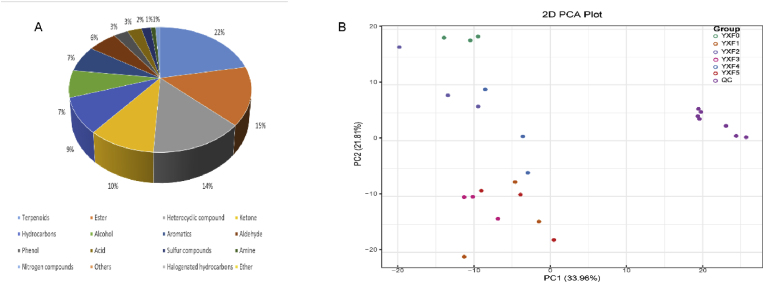
Metabolite categories and PCA score Figure. (A)Type and proportion of volatile metabolites of cigar smoke. Each color represents a metabolite category, and the color block area indicates the high or low proportion of that category. (B)Principal component analysis (PCA) scores of samples and quality control samples at different stages of fermentation. PC1 represents the first principal component, PC2 represents the second principal component, and percentage represents the interpretation rate of this principal component to the data set. Each point in the diagram represents a sample, and samples in the same Group are represented by the same color, and group is the grouping. (For interpretation of the references to color in this figure legend, the reader is referred to the Web version of this article.)

In the process of cigar tobacco leaves fermentation, a total of 613 volatile metabolites were detected based on the GC-MS detection platform and self-built database, which were divided into 16 categories, including terpenes, esters, aldehydes and so on (Fig. 1A); among these compounds, 137 kinds of terpenes were the most prolific, accounting for 22%, followed by 90 kinds of esters, accounting for 15%, and 87 kinds of heterocyclic compounds, accounting for 14%. Ethers, halogenated hydrocarbons, amines, nitrogen-containing compounds and other substances were less abundant. During the whole fermentation process, the volatile substances in cigar tobacco varied and showed an increasing trend. There were significant differences in metabolites in each fermentation cycle, and with the extension of fermentation time, the significant differences in metabolites gradually increased. In general, the fermentation process degrades macromolecular substances that are not conducive to the quality of tobacco leaves, and produces many terpenes, aldehydes and other substances that determine the typical flavor of cigar tobacco.

A multivariate statistical analysis of all identified metabolites was performed in this study. As shown in (Fig. 1B), the PCA score chart of unsupervised multivariate analysis, cigar with different degrees of fermentation showed obvious clustering and differentiation, and there were significant differences between and within sample groups. The QC distribution points of quality control samples were close, which indicates that the system is stable, and the detection quality is reliable. The first two main components accounted for 33.96% (PC1) and 21.81% (PC2) of the total variance. The distance between YXF0 and YXF5 was the largest, indicating that the chemical difference between them was the largest, and the principal component of the sample changed greatly before and after fermentation, and there were more terpenes, esters and aldehydes in YXF5, which affected the flavor of cigars. There were also differences in the principal components of samples from each group, among which YXF2 and YXF4 exhibited the largest differences.

### 3.2 Differential metabolites selected in cigar tobacco leaves

**Fig. 2:**
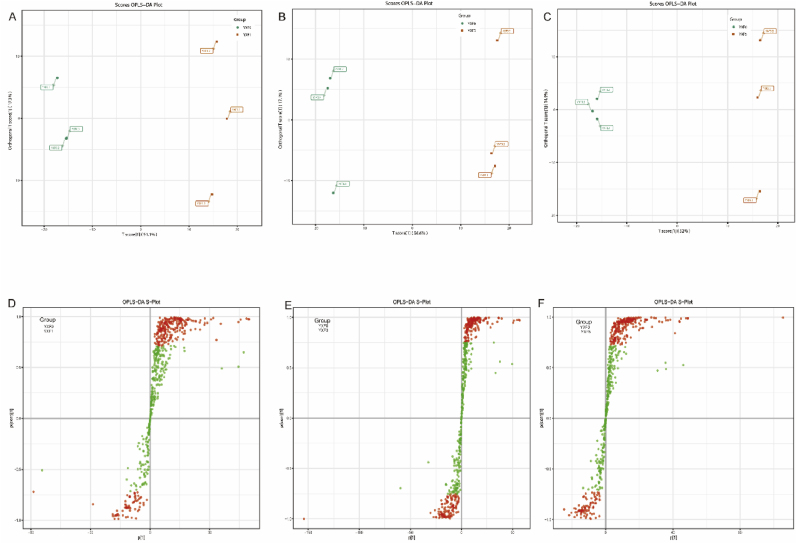
OPLS-DA model generated fermentation in different periods of the score map and volcano map. (A)YXF0 vs. YXF1 OPLS-DA score chart. (B) YXF0 vs. YXF3 OPLS-DA score chart. (C)YXF0 vs. YXF5 OPLS-DA score chart. Each point in the diagram represents a sample, and samples in the same Group are represented by the same color. (D) YXF0 vs. YXF1 OPLS-DA *S*-plot chart. (E) YXF0 vs. YXF3 OPLS-DA *S*-plot chart. (F) YXF0 vs. YXF5 OPLS-DA *S*-plot chart. Red dots indicate that these metabolites have VIP values greater than or equal to 1, and green dots indicate that these metabolites have VIP values less than 1. (For interpretation of the references to color in this figure legend, the reader is referred to the Web version of this article.)

To further determine the differences in major volatile metabolites at different fermentation stages, an OPLS-DA model was established (Fig. 2A B C). PLS-DA is a multivariate statistical analysis method with supervised pattern recognition, which can maximize the distinction between groups and is conducive to finding differential metabolites. OPLS-DA combines orthogonal signal correction (OSC) and PLS-DA methods, which can decompose X matrix information into two types of information related to Y and not related to Y, and screen differential variables by removing irrelevant differences. Based on the OPLS-DA results, VIP of OPLS-DA model obtained by multivariate analysis can initially screen out metabolites with differences between different varieties or tissues. VIP ≥1 metabolites showed significant difference. R^2^X and R^2^Y represent the interpretation rate of the built model for the X and Y matrices, respectively, and Q^2^ represents the prediction ability of the model. The closer the three indices are to 1, the more stable and reliable the model is, and Q^2^ > 0.9 is an excellent model (Table 1).

**Table 1:** Interpreted rates and predictive power of the OPLS-DA models between different sample groups.

Group	R^2^X	R^2^Y	Q^2^
YXF0 vs YXF1	0.684	0.995	0.912
YXF0 vs YXF3	0.736	0.999	0.966
YXF0 vs YXF5	0.67	1	0.943

Notes: R^2^X and R^2^Y represent the interpretation rate of the built model for X and Y matrix respectively, and Q^2^ represents the prediction ability of the model. The closer the three indicators are to 1, the more stable and reliable the model is. Q^2^ > 0.5 can be considered as an effective model, and Q^2^ > 0.9 is an excellent model.

The differences screened out in the fermentation process are shown in Table 2, and there are significant differences between the groups, indicating that there are significant differences in the differential metabolites in different fermentation stages. A total of 263 differential metabolites were detected during the fermentation process. Terpenes were the most abundant (60), followed by esters (34). After fermentation, the total metabolites with significant differences increased, and the number of YXF0 vs YXF3 decreased most (76). YXF0 vs YXF5 group had the most upregulated metabolites (135). YXF0 vs YXF5 group had the highest number of metabolites (187). In the YXF0 vs. YXF1 group, the downregulated differential metabolites included 14 terpenes, such as β-pinene, 3,7,11-trimethyl-1,6,10-dodecatriene-3-ol. The upregulated differential metabolites mainly included 23 terpenes such as 1,3,6,10-Cyclotetradecatetraene, 3,7,11-trimethyl-14-(1-methylethyl)-, [*S*-(E,Z,E,E)]-, 16 esters, 8 acids such as 3-hydroxy-3-methyl-butyric acid, 2,3-dihydroxy-benzoic acid, 8 aldehydes, such as 2-hydroxybenzaldehyde and 4-hydroxybenzaldehyde, and 7 alcohols, such as 4-hydroxy-phenyl ethanol (Fig. 2D). Among the samples in the YXF0 vs. YXF3 group, the downregulated differential metabolites included 24 terpenes, such as α-pinene, l-carvone, 10 esters such as geranyl butyrate, 2 phenols, such as p-cresol and 2-ethyl-phenol, and 2 acids, such as cinnamic acid. Upregulated differential metabolites included 24 terpenes, such as 1,3,6,10-Cyclotetradecatetraene, 3,7,11-trimethyl-14-(1-methylethyl)-, [*S*-(E,Z,E,E)]-, 15 heterocyclic compounds, and 7 acids, such as 3-hydroxy-3-methyl-butyric acid, benzoic acid, and palmitoleic acid (Fig. 2E). In the YXF0 vs. YXF5 group, the upregulated differential metabolites included 29 terpenes, 21 heterocyclic compounds, 8 acids, such as 3-hydroxy-3-methyl-butyric acid, adipic acid and palmitoleic acid, and 6 alcohols, such as 4-hydroxy-phenyl ethanol. Downregulated differential metabolites included 19 terpenes, such as (E)-farnesol and 7 esters, etc. (Fig. 2F). The above results showed that the quantity of volatile differential metabolites was large in the fermentation process of cigar tobacco leaves, and the quantity and type of differential metabolites changed constantly with the fermentation process, and the upregulated differentiators included benzaldehyde, 1,3,6,10-Cyclotetradecatetraene, 3,7,11-trimethyl-14-(1-methylethyl)-, [*S*-(E,Z,E,E)]- and other flavor-causing substances.

**Table 2:** Statistics of differential metabolite numbers.

group name	All sig diff	down regulated	up regulated
YXF0 vs YXF1	155	33	122
YXF0 vs YXF2	51	17	34
YXF0 vs YXF3	186	76	110
YXF0 vs YXF4	101	37	64
YXF0 vs YXF5	187	52	135

Notes: group name:Differential comparison of the group information; All sig diff: Number of significant metabolites; down regulated: Downregulated metabolite number; up regulated: Upregulated the metabolite number.

### 3.3 Analysis of differential metabolites in cigar tobacco leaves

**Fig. 3:**
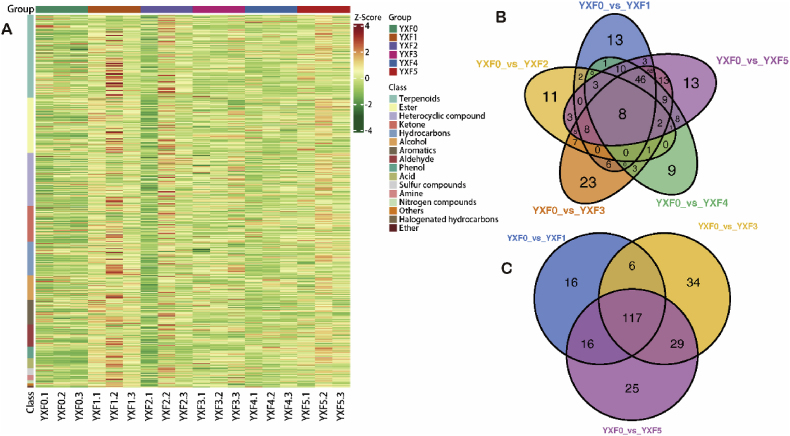
Analysis of cluster heat map and Venn diagram for different fermentation periods. (A) Sample population clustering diagram. Horizontal is the sample name, vertical is the metabolite information, Group is the group, and different colors are the colors filled with different values obtained after standardized treatment of different relative contents (red represents high content, green represents low content). Where, Class is the first-class classification of substances; The cluster line on the left of the figure is the metabolite cluster line, and the cluster line on the top of the figure is the sample cluster line. (B) Overall sample Venn diagram. (C) YXF0 vs. YXF1, YXF0 vs. YXF3, YXF0 vs. YXF5 group difference Venn diagram. Each circle in the figure represents a comparison group, and the numbers in the overlapping part of the circle and the overlapping part represent the number of different metabolites shared between the comparison groups, while the numbers in the non-overlapping part represent the number of different metabolites unique to the comparison group. (For interpretation of the references to color in this figure legend, the reader is referred to the Web version of this article.)

**Fig. 4:**
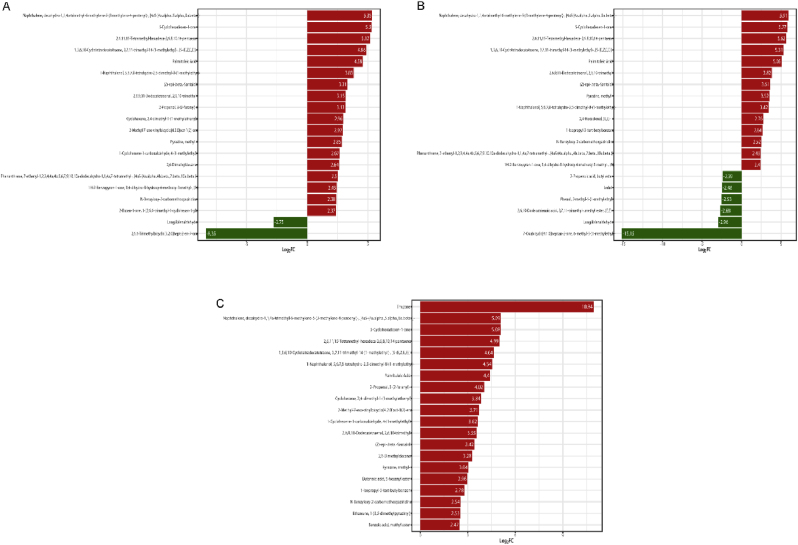
The top 20 metabolites bar chart in the comparison of the difference multiples in each group. (A)YXF0 vs. YXF1 group. (B)YXF0 vs. YXF3 group. (C)YXF0 vs. YXF5 group. The horizontal coordinate is the log_2_FC of the differential metabolite, that is, the logarithmic value of the differential multiple of the differential metabolite is taken as the base 2, and the vertical coordinate is the differential metabolite. Red represents upregulated metabolite content and green represents downregulated metabolite content. (For interpretation of the references to color in this figure legend, the reader is referred to the Web version of this article.)

The cluster heatmap analysis of the selected metabolites with significant differences, as shown in (Fig. 3A), can more clearly reflect the dynamic changes in the differential metabolites in cigar tobacco leaves at different fermentation stages. The differences in volatile compounds between groups are significant, and the similarities and differences in volatile metabolites between different sample groups at different fermentation times. The results showed that YXF5 terpenoids, esters, ketones and heterocyclic substances increased, while alcohol and phenol decreased. Second, we evaluated the shared and unique volatile differential metabolites in different fermentation cycles using a Venn diagram (Fig. 3B) to better visualize the overlap of differential metabolites and excavate important differential metabolites. There were 8 kinds of common differential metabolites in the whole fermentation process of cigar tobacco leaves, including 2 terpenes, 2 aromatics, 2 ketones, 1 ester and 1 heterocyclic compound, all of which tended to be upregulated. Eleven terpenes, including α-pinene and β-pinene, were downregulated at the late stage of fermentation. YXF0 vs. YXF1, YXF0 vs. YXF3, and YXF0 vs. YXF5 had 117 different metabolites in common (Fig. 3C). The common upregulated differential metabolites in the three groups were 3-hydroxy-3-methyl-butyric acid, benzoic acid, 4-hydroxybenzaldehyde, 4-hydroxy-phenylethanol, palmitoleic acid and 1,3,6,10-Cyclotetradecatetraene, 3,7,11-trimethyl-14-(1-methylethyl)-, [*S*-(E,Z,E,E)]-, while the common downregulated differential metabolites were.beta.-Myrcene and geranyl butyrate. The common upregulated differential metabolites all influence the formation of cigar flavor to some extent. To better understand the changes in key differential metabolites at each stage, the top 20 substances with the largest fold change in each group were selected (Fig. 4). Among YXF0 vs. YXF1, there were 18 upregulated metabolites, including 6 terpenes, 4 hydrocarbons, 4 heterocyclic compounds, 2 ketones, 1 acid and 1 phenol. Downregulated metabolites included two terpenoids and two ketones (Fig. 4A). In YXF0 vs. YXF3, there were 14 upregulated metabolites, including 5 terpenes, 3 heterocyclic compounds, 1 ketone, hydrocarbon, acid, phenol, aldehyde and aromatic compounds, and 3 downregulated terpenes, 2 esters and 1 ketone (Fig. 4B). In YXF0 vs. YXF5, the top 20 metabolites with the largest changes in multiples all showed an upregulated trend, including 6 terpenes, 4 hydrocarbons and 4 heterocyclic compounds, 2 esters, and 1 ketone, phenol, acid and aromatic compound (Fig. 4C). Among the top 20 substances with the greatest changes, upregulated substances accounted for most of them, especially the top 20 substances with the greatest changes in the later period were upregulated substances, and upregulated substances had the most terpenes. These include alpha-Thujone、1,3,6,10-cyclotetradecatetraene,3, 7,11-trimethyl-14-(1-methylethyl)-, [*S*-(E,Z,E,E)]-, etc.

### 3.4 Exploration of metabolic pathways

**Fig. 5:**
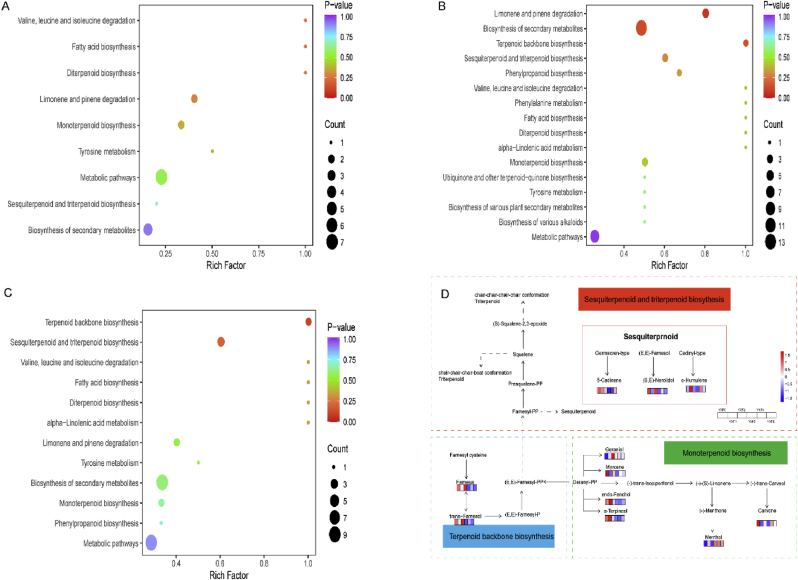
KEGG enrichment pathway map of different metabolites in different fermentation cycles. (A) YXF0 vs. YXF1 enrichment map. (B) YXF0 vs. YXF3 enrichment map. (C) YXF0 vs. YXF5 enrichment map. The horizontal coordinate represents the Rich Factor corresponding to each path, the vertical coordinate is the path name (sorted by *P*-value), the color of the points reflects the *P*-value size, and the redder indicates the more significant enrichment. The size of the dots represents the number of differentiated metabolites enriched. (D) Integration map of major metabolic pathways during fermentation. (For interpretation of the references to color in this figure legend, the reader is referred to the Web version of this article.)

To further determine the possible role of differential metabolites in flavor formation, these differential metabolites were annotated in the KEGG database, and the pathways of significant enrichment in different sample groups are shown in (Fig. 5A B C). The main pathways of differential metabolism include terpene stem biosynthesis, sesquiterpene and triterpene biosynthesis, degradation of valine, leucine and isoleucine, fatty acid biosynthesis, diterpene biosynthesis, linolenic acid metabolism, etc. In general, the differentially enriched metabolites of amino acid degradation metabolism such as valine, leucine, isoleucine, tyrosine, phenylalanine, linolenic acid and phenylpropanoid biosynthesis annotation were more abundant, suggesting that amino acid metabolism and phenylpropanoid synthesis may provide upstream substances for the generation of various flavor metabolites. Determines the metabolic basis of fermentation process and the formation of cigar aroma substances beta.-Myrcene、 1,3,6,10-Cyclotetradecatetraene, 3,7,11-trimethyl-14-(1-methylethyl)-, [*S*-(E,Z,E,E)]-、(E)-farnesol and 2,6,10-Dodecatrienal, 3,7,11-trimethyl-, (E,E)- were noted in the biosynthetic pathways of monoterpenes, diterpenes, sesquiterpenes and triterpenes, respectively. Terpenes mostly have aroma, which determines the formation of flavor during cigar tobacco leaves fermentation.

## 4 Discussion

Tobacco aromatic substances can be divided into ketones, aldehydes, acids, esters, alcohols and alkanes according to their aromatic functional groups. Metabolomics studies have shown that fermentation greatly impacts volatile metabolites in cigar tobacco leaves, and terpenes account for the largest proportion of volatile compounds in the fermentation process. In the KEGG pathway annotated by significantly different metabolites, terpene-related pathways are as follows: terpenoid backbone biosynthesis, monoterpenoid biosynthesis, diterpenoid biosynthesis, sesquiterpenoid and triterpenoid biosynthesis. The biosynthesis pathway of sesquiterpenoids and triterpenoids has a great influence on cigar flavor during cigar tobacco leaves fermentation. Most terpenoids have aroma or aroma precursors, such as solanone and solanedione, which are degradation products of 1,3,6,10-cyclotetradecatetraene,3, 7,11-trimethyl-14-(1-methylethyl)-, [*S*-(E,Z,E,E)]-, have light, pleasant and slightly sweet aroma. Therefore, further integrated analysis of the anabolic pathways of terpenoids in the fermentation process was performed, as shown in (Fig. 5D). (E)-farnesol、2,6,10-Dodecatrienal, 3,7,11-trimethyl-, (E,E)- and important synthetic precursors of terpenes are generated in the biosynthesis pathway of the terpene skeleton, and (2E,6E)-farnesyl diphosphate is generated under the action of various enzymes and then enters the sesquiterpene and triterpene biosynthesis pathways. In addition, the terpene skeleton biosynthesis pathway leads to geranyl-PP, the precursor of monoterpene biosynthesis. The main changes in volatile metabolites in the sesquiterpene and triterpene biosynthesis pathways involved sesquiterpenoids. Sesquiterpenoids are mainly distributed in plants and exist in volatile oils in various forms. They are important aroma producing substances, most of which have strong aroma. 1,6,10-Dodecatrien-3-ol, 3,7,11-trimethyl-、*trans*-Farnesol、2,6,10-Dodecatrienal, 3,7,11-trimethyl-, (E,E)- with sweet、floral、green fragrance, which greatly affects the flavor of the cigar.

Second, in the classification of metabolic pathways involving differential metabolites, the synthesis and degradation of various amino acids accounted for a large proportion of metabolic pathways. Amino acids are important nitrogenous compounds in tobacco products and are also among the main synthetic raw materials of aroma substances. Among carboxylic acids and amino acids, low molecular weight carboxylic acids give smoke odor and aromatic characteristics, and high molecular fatty acids make the smoke soft and palatable. Among them, 3-methylvaleric acid and isovaleric acid are characterized by flavor, cheese and fruit, while phenylacetic acid can give sweet and honey-like flavor to tobacco. Yu Lianying [26] performed a metabolomics analysis, through the semiquantitative analysis of important metabolites, and showed that the influence of amino acid metabolic pathways in the entire metabolic process of cigar accounted for a considerable proportion. Amino acids and their related metabolites are widely distributed in various amino acid metabolic pathways and change with the progress of fermentation. These include phenylalanine, valine, leucine, isoleucine and tyrosine. Phenylalanine metabolism is among the most important secondary metabolic pathways in plants and is mainly divided into the phenylpropane metabolic pathway and flavonoid synthesis pathway [27]. The phenylalanine biosynthesis pathway is a biosynthesis pathway that occurs in plants, through which plants can synthesize many important compounds, such as flavonoids, coumarins and lignin. Phenylalanine metabolite benzaldehyde has almond aroma, cherry aroma and sweet aroma, phenylacetaldehyde has rose fragrance, benzyl alcohol and benzyl alcohol have mellow aroma, these substances have obvious influence on the formation of cigar aroma style [28]. In addition, research shows that Mont-4 and Changcheng2 mainly contain a series of volatile fragrance components, such as 6-methyl-5-hepene-2-one, benzaldehyde, limonene, n-hexanal and 3,5-dimethylphenol [29]. Benzaldehyde and 3,5-dimethylphenol were also detected in the fermentation process of cigar tobacco leaves, among which benzaldehyde exhibited bitter almond, cherry and nut aromas; 3,5-dimethylphenol exhibited a smoky aroma and is an important volatile component of tobacco. Most tobacco aroma substances are produced by secondary metabolism. The amino acid metabolism pathway, phenylpropanoid biosynthesis and terpenoid anabolism explored in the fermentation process all affect the flavor of cigar tobacco leaves and determine the quality of tobacco leaves.

From the perspective of the contribution of volatile metabolites to the flavor and quality of cigar tobacco leaves during fermentation, OPLS-DA was used to excavate key differential metabolites at different stages of fermentation and explore the main enrichment pathways during fermentation, revealing the internal mechanism of the effect of fermentation on the flavor of cigar tobacco leaves, and providing scientific basis for optimizing the fermentation technology of high-quality cigar tobacco leaves on the whole. It lays a solid foundation to produce of high-quality cigar materials. However, the interaction between the terpenoid pathway, phenyl-C pathway and amino acid degradation pathway in cigar tobacco leaves fermentation has not been thoroughly studied in this study. Future research can further study the relationship between volatile metabolites and various metabolic pathways during cigar tobacco leaves fermentation, to deepen the development of cigar flavor.

## 5 Conclusions

In the process of fermentation, 613 kinds of volatile metabolites were detected in Yunxue1 tobacco leaves from Yuxi Chengjiang, 263 kinds of metabolites with significant differences were detected, among which, benzaldehyde with nut-fragrance, bean fragrance and spice fragrance was the main difference metabolite. There are also the precursor substances 1,3,6,10-cyclotetradecatetraene,3, 7,11-trimethyl-14-(1-methylethyl)-, [*S*-(E,Z,E,E)]- that can produce solanone and solanedione with light, pleasant and slightly sweet aroma. In addition, in the fermentation process, the biosynthesis pathway of monoterpene, diterpene, sesquiterpene and triterpene, which are the main enrichment of key differential metabolites, contributes to the sweetness and fragrance of cigar. The metabolism pathway of amino acid degradation of valine, leucine, isoleucine and biosynthesis of phenylpropane determines the formation of flavor in cigar fermentation. It provides a deeper theoretical basis for the promotion of cigar tobacco leaves fermentation technology, and provides better raw materials for cigar tobacco market, to promote the promotion of the international status of cigar.

## Author contribution

Conceptualization, F.L. and G.Z. (Guanghai Zhang); methodology, H.Y.; software, G.Z (Gaokun Zhao); validation, J.F; investigation, G.Z (Guanghai Zhang); data curation, Y.W; writing—original draft preparation, J.F; writing—review and editing, F.L and G.K.; funding acquisition, G.K. All authors have read and agreed to the published version of the manuscript.

## Declaration of competing interest

There are no conflicts of interest in the submission of this manuscript, and manuscript is approved by all authors for publication.

## Data Availability

Data will be made available on request.
